# Connecting the Dots in Emerging Mast Cell Research: Do Factors Affecting Mast Cell Activation Provide a Missing Link between Adverse COVID-19 Outcomes and the Social Determinants of Health?

**DOI:** 10.3390/medsci10020029

**Published:** 2022-05-28

**Authors:** Rachel da Silveira Gorman, Iffath Unissa Syed

**Affiliations:** 1 School of Health Policy and Management, York University, 4700 Keele St., Toronto, ON M3J 1P3, Canada; 2 Health Policy and Administration, The Pennsylvania State University, 47 Shenango Ave, Sharon, PA 16146, USA; ixs5254@psu.edu

**Keywords:** COVID-19, mast cells, social determinants of health, autoimmune disease, chronic illness, adverse vaccine effects, MCAS, long COVID, environmental illness, cytokine storm, neurological inflammation

## Abstract

Evidence continues to emerge that the social determinants of health play a role in adverse outcomes related to COVID-19, including increased morbidity and mortality, increased risk of long COVID, and vaccine adverse effects. Therefore, a more nuanced understanding of the biochemical and cellular pathways of illnesses commonly associated with adverse social determinants of health is urgently needed. We contend that a commitment to understanding adverse outcomes in historically marginalized communities will increase community-level confidence in public health measures. Here, we synthesize emerging literature on mast cell disease, and the role of mast cells in chronic illness, alongside emerging research on mechanisms of COVID illness and vaccines. We propose that a focus on aberrant and/or hyperactive mast cell behavior associated with chronic underlying health conditions can elucidate adverse COVID-related outcomes and contribute to the pandemic recovery. Standards of care for mast cell activation syndrome (MCAS), as well as clinical reviews, experimental research, and case reports, suggest that effective and cost-efficient remedies are available, including antihistamines, vitamin C, and quercetin, among others. Primary care physicians, specialists, and public health workers should consider new and emerging evidence from the biomedical literature in tackling COVID-19. Specialists and researchers note that MCAS is likely grossly under-diagnosed; therefore, public health agencies and policy makers should urgently attend to community-based experiences of adverse COVID outcomes. It is essential that we extract and examine experiential evidence of marginalized communities from the broader political–ideological discourse.

## 1. Introduction

In most cases, patients who are infected with the novel SARS-CoV-2 (COVID-19) are asymptomatic or only present with mild to moderate symptoms, including fever, dry cough, shortness of breath, loss of smell or taste, and/or gastro-intestinal symptoms. Nevertheless, some patients develop more severe diseases, including pneumonia [1], and have a significant risk of further morbidity and mortality. Research continues to emerge that COVID-19 can affect many organ systems [2] and can precipitate autoimmune disease [3,4]; however, the underlying mechanisms remain unclear. Furthermore, since the early days of the pandemic, correlations between social inequities and higher instances of mortality and morbidity have been reported [5,6].

COVID-19 patients presenting with diabetes, obesity, hypertension, smoking, and lung disease are considered to be at high risk of developing severe disease [7,8]. In the literature on the social determinants of health, the incidence and distribution of these pre-existing conditions have long been associated with socioeconomic disparity [9,10].

Throughout the pandemic, social determinants of health (SDH) such as poverty, physical environment, and race have impacted disease outcomes [11]. SDH include housing, workplace stress, income, education, and access to food; gender, race, and other categories of social exclusion are also considered as cross-cutting social determinants of health [12]. Recent research suggests gender differences impact COVID-19 outcomes [13,14], as do age differences such as the disproportionate impact on infants and very old/frail adults [15]. Other work found a strong correlation between SDH and racial disparities in COVID-19 mortality [16].

Clinical practice tends to be slow to adopt new scientific research and to change the standards of care accordingly. The intensive real-time global focus of medical research on COVID-19 provides an opportunity to investigate cellular mechanisms of health inequities, which may, in turn, provide fruitful new directions for health research and public health policy.

There is a growing body of literature implicating mast cells in many chronic illnesses, including allergies and sensitivities, inflammatory disorders, and tumors [17]; Alzheimer’s and neurodegenerative disease [18]; depression [19]; obesity and low-grade inflammation [20]; asthma [21]; multiple sclerosis [22]; gastrointestinal disease [23]; and cardiovascular disease [24]. This growing list of chronic illnesses in which mast cells are implicated resembles the list of chronic illnesses in which SDH as well as environmental factors such as air pollution are implicated.

In this article, we synthesize emerging literature on mast cells in order to hypothesize a link between diseases commonly associated with SDH, and increased COVID-related morbidity and mortality. We further propose that new learnings on mast cell disease can contribute to pandemic recovery, and new lines of inquiry in global public health, health policy, and health equity.

## 2. Implication of Mast Cells in Chronic Illness

Mast cells are tissue-bound cells of the immune system that originate from hematopoietic stem cells in the bone marrow [25,26]. They mature in the interstitial tissue of most organs [25] and are prevalent in the skin, gastrointestinal tract, and respiratory tract, where they are relatively long-lived as mature cells. Despite some research focusing on the role of mast cells in systemic sclerosis [27,28], early mast cell research focused on mast cell involvement in immune responses to pathogens [29] and their role in allergy and anaphylaxis [30].

More recently, mast cells have been described as playing a key role in hypersensitivity reactions, chronic inflammatory and autoimmune disorders, auto-immune thyroid disease [31], multiple sclerosis, rheumatoid arthritis, and insulin-dependent type II diabetes mellitus [26]. Mast cells are activated in eosinophilic esophagitis, and may correlate with acid exposure [32], and poor outcomes in solid and hematologic tumors [33]. Mast cells also play a role in tissue remodeling, and in cancers [34]. Increased mast cells have been observed in the vascularization of new and aggressive tumors [35]. Recent discoveries show that mast cells develop phenotypic differences based on tissue residency, leading to specific mast cell responses in different organ systems [36].

Mast cell hyperactivity, sometimes known as Mast Cell Activation Syndrome (MCAS), is a chronic multisystem disease that causes inflammatory, allergic, and dystrophic issues [37]. Mast cell hyperactivity has been demonstrated in fibrosis of the lungs, liver, kidneys [1], and heart ([25]), as well as epiploic appendagitis of the large intestines [38]. Furthermore, using regression analysis of validated chemical sensitivity scoring of patients with MCAS, patients reporting chemical intolerance, and a control group, researchers hypothesize that mast cell activation may explain chemical sensitivity [39].

### Social and Environmental Bases of Mast Cell Disorders

Workplace exposure to stress, heavy machinery, and mechanization may be pathways for mast cell-driven chronic illness. Researchers demonstrate that rats kept in a high-traffic area of the lab had greater mast cell degranulation and tissue remodeling in their intestinal mucosa, compared to rats kept in a low-traffic area [40]. Others propose a model of vibration-induced mast cell degranulation based on studies of vibratory urticaria [41].

Exposure to air pollution may be a pathway for mast cell-driven chronic illness. A mouse model demonstrates that fine particulate matter facilitates IgE-mediated mast cell degranulation and increased cytokine expression [42]. Investigating the cytotoxic effects of water-soluble extracts from fine and coarse particulate matter on mast cell lines, others show that both quantity and quality of particulate are implicated in severe tracheobronchial and alveolar illness [43].

Smoking-related lung diseases have well-described mast cell activation and mast cell-induced signaling cascades [44]. Understanding the mechanisms involved in cigarette-related mast cell activation may help us develop therapies for fossil fuel emission particulate matter-induced pulmonary and systemic illness [44].

Exposure to water- and food-borne environmental contaminants may be a pathway for mast cell-driven chronic illness. For example, environmental estrogens can induce mast cell degranulation [45]. Furthermore, mast cell degranulation correlates with cytotoxicity of chemical pollutants in e-waste soil-derived extracts from sites in Ghana, Benin, and Nigeria [46]. Furthermore, these samples differed significantly from control soils. Researchers investigated the effect of three persistent organic compounds (POPs) on mouse models of mast cell-mediated inflammatory responses [47]. They found the three POP compounds caused a greater mast cell response than bisphenol A (BPA). Importantly, they hypothesize that POPs as widely distributed food contaminants may be contributing to rising rates of immune dysfunction.

## 3. Mast Cells and the COVID-19 Cytokine Storm

COVID-19 can activate mast cells that are found in the respiratory tract in the initial stage of infection [8]. Although mast cell activation can be helpful in fighting infections, extensive mast cell activation leads to the release of inflammatory cytokines and chemokines, which further worsen inflammation and increase the severity and likelihood of mortality from COVID-19 ([8,48,49,50]). The virus activates mast cells to release several pro-inflammatory molecules, including histamine, tryptase, IL-1β, CCL2, IL-6, GM-CSF, and TNF-α, which are implicated in COVID-19 disease ([8,49,50,51,52,53]). Accordingly, mast cell activation in the respiratory tract can worsen lung inflammation and lung failure from COVID-19 [8].

Mast cells are present in higher concentrations in localized tissues during chronic disease [54]. Since people experiencing chronic disease, including chronic conditions often associated with adverse social determinants, have higher concentrations of mast cells in specific tissues, we speculate that some people may have more target cells where the SARS-CoV-2 virus can enter.

### Involvement of Mast Cells vs. Other White Blood Cells in COVID-19 Cytokine Release

Acute respiratory distress syndrome (ARDS) associated with severe COVID-19 is characterized by elevated levels of inflammatory cytokines such as: IL-1β, IL-2, IL-4, IL-6, IL-10, IFN-γ, and TNF-α; as well as “profound alterations in cell populations associated with the immune response against viruses, such as monocytes, macrophages, neutrophils, NK cells, B lymphocytes, CD8+ T lymphocytes, and memory and regulatory CD4+ lymphocytes” [55]. While mast cells are leukocytes derived from hematopoietic precursors, they are “tissue resident distributed throughout the body and abundantly found along the respiratory tract” [55] as well as the gastrointestinal tract, connective tissue, and skin.

Soria-Castro et al. [55] demonstrate that serum carboxypeptidase-A3 was more reliable than serum serotonin for detecting severe COVID-19 disease, with similar predictive values to C-reactive protein, which suggests a relationship between mast cell activation and severe COVID-19 [55]. Mast cells are associated with tissue damage induced by an excessive inflammatory response during viral infections. Mast cell-deficient mice show less lung damage than wild-type mice when infected with the influenza A virus [55] Soria-Castro et al. (2021). Further, the mast cell-deficient mice had “a decreased production of TNF-α, CCL2, CCL3, CCL4, CXCL2, and CXCL10, suggesting a crucial role of MC in the ‘cytokine storm’ triggered by influenza infection” [55].

## 4. Involvement of Mast Cells in the Pathogenesis of COVID-19

Atiakshin et al. [56] describe mast cells as “migrating unicellular glands that regulate local homeostasis under both normal and pathological conditions” [56]. As proinflammatory effector cells of the immune system, mast cells play an active role in various infectious diseases, including bacterial and viral infections [57]. During infections, mast cells often become activated and release various pro-inflammatory mediators [57]. Mast cells express coronavirus receptors such as CD26, and they are hypothesized to contribute to coronavirus-mediated inflammation in the lung [57].

Inflammatory mediators from mast cell activation are associated with severe COVID-19 [58]). By measuring mast cell-specific protease chymase, [58] Tan et al. (2021) observed widespread degranulation of mast cells during airway inflammation in SARS-CoV-2-infected mice. They also confirmed widespread mast cell activation in COVID-19-infected primates by detecting heparin-containing granules in the lung tissue through fluorescence staining [58]. Tan et al. confirmed mast cell activation in humans by correlating plasma chymase levels with COVID-19 disease severity [58].

Furthermore, evidence of mast cell activation in the lungs post infection, after the patients had negative PCR tests “could suggest a role for mast cells in the sustained inflammatory response that limits disease resolution” [58].

Tan et al. [58] note that the intravascular coagulation, endothelial damage, damaged microvasculature, and increased incidence of myocardial infarction experienced by some patients with severe COVID-19 are consistent with the effects of mast cells in other sterile inflammatory conditions. They further observe that mast cells “line the blood vessels within tissues, which not only places them in a location where they can directly exert their effects on the vasculature, but also where their mediators can gain access to the blood” [58].

### Mast Cell Degranulation, Transgranulation, De Novo Mediator Synthesis, and COVID-19

One mode by which mast cells could influence a given inflammatory condition is by effects attributed to compounds that are released when mast cells degranulate, leading to a “massive release of the preformed mediators that are stored within the mast cell secretory granules” [59]. In addition to mediator release through degranulation, mast cells may release specific inflammatory mediators from pre-formed pools, or synthesize them do novo [59].

Atiakshin et al. [56] note that “gradual degranulation may be involved in the cooperation of MCs with each other, coordinating the level of their secretory activity in normal or pathological conditions, in particular, chronic inflammation, allergies, urticaria, Crohn’s disease, and oncogenesis.” They further note that “transgranulation between MCs and fibroblasts, capillary endothelium, and neurons has been described” [56].

Tan et al. [58] suggest that it remains to be shown whether mast cells can be activated by autoantibodies in the absence of an active infection, which “might be relevant to long COVID-19 with persistent symptoms” [58].

## 5. Mast Cell Proteases and the Pathogenesis of Chronic Diseases and COVID-19

In a 2022 review of mast cell disorders, Leru [60] notes that mast cells “contain a plethora of active mediators, such as biogenic amines (histamine, serotonin), serines and other proteases (tryptase, serine S1, chymase, cathepsin G, granzyme, carboxypeptidase), lysosomal enzymes, and proteoglycans (heparin, chondroitin sulfates).” So far, the roles of mast cell proteases tryptase, chymase, and carboxypeptidase A3 in COVID-19 pathogenesis have been described.

### 5.1. Tryptase

Tryptase secretion from both connective tissue- and mucosa-resident MCs can trigger the secretion of IL-8 and IL-6 from human eosinophils, and recruit neutrophils to affected sites [61]. Furthermore, mast cell-derived tryptase “induces the expression of inflammatory cytokines, such as IL-1β and IL-8 from endothelial cells in a dose-dependent manner” [61]. Tryptase has also “been shown to have epigenetic effects due to its influence on the state of nuclear histones and DNA stabilization” [56].

Karimi et al. [61] report that serum tryptase can predict COVID-19 severity. Tryptase is associated with vascular leakage and may contribute to COVID-19 brain fog due to its ability to increase the permeability of the blood–brain barrier [61]. Tryptase may also contribute to post-COVID-19 pulmonary fibrosis through triggering “the migration and proliferation of lung fibroblasts resulting in airway remodeling” and upregulating fibroblast collagen synthesis [61].

### 5.2. Chymase

The internalization of the SARS-CoV-2 virus leads to the reduction in angiotensin 1 converting enzyme 2 (ACE 2) receptors on the cell surface, which leads to a decrease in angiotensin II degradation and the generation of angiotensin 1–7 [62]. There is not yet a consensus on the mechanisms by which the accumulation of angiotensin II and/or the reduction in ACE 2 impact COVID-19 outcomes. Because ACE2 is low in “common chronic pathologies including hypertension, angiocardiopathy, type 2 diabetes, chronic renal failure, pulmonary diseases and liver diseases. Several authors proposed that the severity of Covid-19 is exacerbated by the degradation of ACE2 by the virus” [63].

Large amounts of mast cell-released chymase are present in a wide range of pathologies [59]. Moreover, the “administration of purified/recombinant chymase in different animal models has been shown to cause the accumulation of various inflammatory cell types, including eosinophils, neutrophils, lymphocytes and macrophages” [59].

Several authors suggest that mast cell-derived chymase may also play a key role in COVID-19, as it is able to generate angiotensin II independently from ACE activity [62]. In pre-COVID research, Myazaki et al. [64] showed that mast cell chymase leads to the conversion of angiotensin I to angiotensin II. Tan et al [58] note that mast cell chymase is a potent converter of angiotensin I to angiotensin II, and that the production of chymase by mast cells is associated with vascular diseases. Evidence of the conversion of angiotensin I to angiotensin II by chymase “has been especially found in morbid tissues following the migration of mast cells” including in heart disease, where chymase inhibitors reduce mortality rates after myocardial infarction [64]. In addition, chymase activates transforming growth factor beta (TGF-β), and matrix metalloproteinases, which are involved in pulmonary fibrosis [65]. 

Konrath et al. [62] find that “chymase inhibitors show reasonable evidence to prevent or reduce the COVID-19 acute inflammation and consequently the mortality rate of infected individuals.” In addition, chymase inhibition blunts the generation of profibrotic transforming growth factor-β [59].

### 5.3. Carboxypeptidase A3

Atiakshin et al. [56] suggest that serum carboxypeptidase A3 (CPA3) is a good biomarker for identifying severe COVID-19 patients. In human mast cells, “chymase is usually coordinately expressed at a 1: 1 molar ratio with CPA3…. CPA3 can also be expressed in tryptase-positive human MCs with no chymase, including in the mucous membrane of the stomach and small intestine” [56]. The expression of the CPA3 gene is found in mast cells and basophilic leukocytes in patients with an allergic history, and CPA3 is involved in the pathogenesis of cancer and inflammatory diseases of the gastrointestinal tract, and respiratory and cardiovascular systems [56].

Serum CPA3 levels are correlated with “circulating neutrophils and CPR, which are associated with an exacerbated inflammatory response during COVID-19” [55]. While the source of this serum CPA3 is unknown, mast cells are the main source, releasing CPA3 after degranulation [54].

CPA3 has a role in the “biogenesis of the fibrous component of the extracellular matrix” and it has therefore been implicated in the remodeling of the extracellular matrix and fibrosis in COVID-19 [56].

## 6. Mast Cells and Long COVID-19

Research suggests that patients who have experienced COVID-19 infection, but who recover, seem to have long-term problems. Long COVID-19 symptoms or “post-COVID syndrome” symptoms refer to persistent respiratory or systemic symptoms such as chest pain, generalized fatigue, and joint pain after recovery from a COVID-19 infection [1].

Pulmonary fibrosis is one of the main problems that develop in long COVID-19, and results in irreversible and decreased pulmonary function [1]. Mast cells play a pivotal role in pulmonary fibrosis [66]. Tumor necrotizing factor (TNF-α) plays a key role in the development of fibrosis [55], and it is stored in mast cells [67], where it is readily released during mast cell degranulation.

In a survey of self-reported symptoms, researchers compared 136 long COVID patients with 136 controls who had never had overt COVID symptoms and 81 patients with mast cell activation syndrome (MCAS) [68]. They found that mast cell activation symptoms were increased in long COVID, and that the symptom profiles of long COVID mimicked those of MCAS.

## 7. Mast Cells in Adverse Vaccination Effects

Although the COVID-19 vaccine has been promising and reduced the severity of the disease, there is a significant proportion of the population that remains unvaccinated. Vaccine uptake has been slow, not without criticism, and also controversial, with vaccine hesitancy due to fear of the unknown/side effects, and exercise of choice and personal freedom. Some individuals cannot be vaccinated due to adverse reactions to vaccine components. A number of individuals have explored their own therapies, including Ivermectin, which have not been proven to be effective.

So far, studies of adverse COVID-19 vaccine events have overwhelmingly focused on reactions immediately following the injection, and the following 48 h. However, vaccines take time to elicit an immune response, and it is therefore important to consider flare-ups of autoimmune conditions that begin between 10 and 14 days after the injection. In a survey of rheumatic disease patients, 117 (10.4%) self-reported a systemic rheumatic disease flare after their first dose of COVID-19 vaccine (N = 1101), and 85 (13.6%) self-reported a systemic rheumatic disease flare after their second dose (N = 626) [69]. We are not aware of any studies of post-vaccine mast cell activation in the days and weeks following COVID-19 injection.

As many mast cell researchers note that MCAS is likely grossly under-diagnosed [70], studies of post-vaccine mast cell activation in the general population would go a long way toward identifying and explaining post-vaccine flares, and would therefore provide science-based reassurance in communities where historically justified mistrust of medical science coupled with anecdotal reports of adverse events has led to vaccine hesitancy.

In one study, researchers hypothesize a mechanism through which adenoviral non-replicating vectors “may trigger aberrant inflammatory and immune responses in people affected by the mast cell activation syndrome (MCAS)” [71] (p. 1) Further, they hypothesize that neo-synthesized spike proteins interacting with the ACE2 receptor on endothelia, platelets, and leucocytes may trigger thrombotic events including vaccine-induced thrombocytopenia.

## 8. Discussion and Potential Therapeutics

### 8.1. Anti-Cytokine Treatments Currently in Use for COVID-19 Infection

The National Health Institute COVID-19 Treatment Guidelines (downloaded 5 August 2022) currently recommend dexamethasone or other corticosteroids only in cases of patients requiring oxygen. There are several immunomodulators under evaluation for COVID-19, including: colchicine; systemic and inhaled corticosteroids; fluvoxamine; granulocyte-macrophage colony-stimulating factor inhibitors; non-SARS-CoV-2 specific immunoglobulins; interleukin-1 inhibitors; interleukin-6 inhibitors; and kinase inhibitors.

Tan et al. [58] note that drugs targeting mast cells and their products may be a promising therapy for preventing severe COVID-19. In a discussion of COVID-19 treatment options for MCAS and mastocytosis, Valent et al. [57] observe that “anti–mediator-type drugs, including antihistamines, antileukotrienes, cromones, and omalizumab (anti-IgE), have been in use for many years, and there is no reasonable evidence to suggest that these drugs exert immunosuppressive effects, even when used over several years.” Hafezi et al. [65] suggest a range of potential drugs for treating COVID-19 cytokine storm including mast cell stabilizing drugs, tryptase inhibitors, zinc-mediated inhibitors, and tryptase-specific antibodies.

### 8.2. Role in COVID-Related Treatment and Pandemic Recovery

Recent in vitro studies involving patch-clamp techniques suggest that certain anti-allergic drugs (olopatadine, ketotifen), particular antibiotics (clarithromycin), and corticosteroids (hydrocortisone, dexamethasone) were all highly effective in stabilizing the deleterious effects of mast cells [1]. Another case report suggests that mast cell stabilizers (quercetin and vitamins C, D, B6, and B12) and naltrexone were effective in the treatment of mast cell-mediated epiploic appendagitis [38]. Mast cell-targeted therapies are also beneficial in the treatment of esophagitis [32]. Because of the role of mast cells in long COVID-19, these therapeutics may be useful in the treatment of post-COVID-19 pulmonary fibrosis and in relieving the symptoms of post-COVID syndrome [1].

It has been proposed that mast cell activity should be modulated in order to treat some of the more adverse effects of mast cell function. While antihistamine plays a central role in mast cell-driven anaphylaxis and allergic reactions, additional pharmacotherapeutics are needed for the treatment of mast cell-related conditions, such as fibrosis to succeed [25]. Instead, it is proposed that mast cell activity can be suppressed either through other pharmacological approaches by directly stabilizing mast cells, or by indirectly inhibiting the chemokines released from mast cells in order to treat or protect against organ fibrosis [1].

Screening and treatment with mast cell stabilizing agents may assist people who are vulnerable to adverse events. For people with diagnosed mast cell disease such as mastocytosis, the current focus is on pre-treatment with antihistamines to avert peri-vaccination anaphylaxis [72]; however, it would be worthwhile to conduct small-scale clinical studies that continue with mast cell stabilization treatment throughout the two-week period post-vaccination, or in some cases, beyond. A longer period of prophylactic treatment seems warranted, considering vaccine science has shown immune activation pathways comprise weeks rather than hours. Robust, science-based attention to post-vaccine flares of chronic illness, and other peri- and post-vaccine adverse events will go far in re-assuring communities where there has been vaccine hesitancy. It is important to consider the concerns of communities who have been historically marginalized in medical research. Qualitative research should be undertaken to identify vaccine hesitancy due to community-based anecdotal evidence. These concerns should be addressed separately from vaccine hesitancy related to political–ideological commitments.

## 9. Conclusions

Although no direct research on mast cells in COVID-19 patients has been performed yet, the prevalence and potential for mast cells to drive substantial morbidity merit epidemiological investigation. Furthermore, inquiry into the role of mast cells in COVID disease presents an opportunity to trace the biochemical and cellular pathways of the social determinants of illness. We suggest that public health experts and medical/healthcare providers should be acquainted with the etiology, pathogenesis, and potential impact of mast cells on patients who are at risk of COVID-19, as well as the general public. Our synthesis of the literature on mast cells, adverse COVID-19 outcomes, and social determinants of health provides a framework for future research to guide policymakers, public health workers, epidemiologists, physicians, and other clinicians in the management and prevention of COVID-19 and other mast cell-mediated illnesses.

## Data Availability

Not applicable.
